# The role of discharge planning services in continuity of care for patients with diabetes: a scoping review

**DOI:** 10.3389/fpubh.2026.1907072

**Published:** 2026-08-19

**Authors:** Xiaotong Han, Ruoxuan An, Simin Zheng, Ling Zhou, Yaxuan Luo, Xuefen Lan, Minhua Chen

**Affiliations:** 1 School of Medicine, Lishui University, Lishui, China; 2 Department of Nursing, The Second Affiliated Hospital of Lishui University, The Second People's Hospital of Lishui, Lishui, China; 3 Department of Endocrinology, The First Affiliated Hospital of Lishui University, Lishui People's Hospital, Lishui, China; 4 Department of Nursing, The First Affiliated Hospital of Lishui University, Lishui People's Hospital, Lishui, China

**Keywords:** continuity of care, diabetes, discharge planning, patient discharge, transitional care

## Abstract

**Background:**

Diabetes has become a major global public health challenge. Increasing pressure on healthcare systems and shorter hospital stays highlight the importance of effective discharge planning in enhancing post-discharge self-management and continuity of care for people with diabetes.

**Aim:**

This scoping review aimed to map existing discharge planning practices that support continuity of care for people with diabetes and to inform the optimization of intervention programs in clinical practice.

**Methods:**

The review was conducted according to the Joanna Briggs Institute (JBI) methodology and reported in accordance with the PRISMA-ScR guidelines. Eligible studies included randomized controlled trials and quasi-experimental studies investigating discharge planning services for adults with type 1 diabetes, type 2 diabetes, or gestational diabetes during the hospital-to-home transition. English and Chinese databases (PubMed, CINAHL, Embase, Web of Science, CNKI, Wanfang, VIP, and SinoMed) were systematically searched for studies published between January 2020 and June 2025. Study selection and data extraction were independently performed by two reviewers. The included studies were synthesized using descriptive and thematic analyses, and methodological quality was assessed using the Cochrane Risk of Bias 2 tool for randomized controlled trials and the JBI critical appraisal checklist for quasi-experimental studies.

**Results:**

A total of 20 studies met the inclusion criteria. Most studies involved adults with type 2 diabetes or mixed populations of adults with type 1 and type 2 diabetes. Technology-enabled components were incorporated into several interventions, whereas explicit theoretical or conceptual foundations were reported in only a subset of studies. Intervention timing varied across studies, with some interventions initiated during hospitalization and continued into the post-discharge period. Delivery approaches included in-person, digital, and hybrid formats. Core components included patient assessment, diabetes education, lifestyle guidance, medication and glucose management, complication prevention, and psychological support. Reported outcomes primarily included HbA1c, self-management-related outcomes, quality of life, and discharge readiness. Outcome assessments were commonly conducted at discharge, whereas post-discharge follow-up assessments were often performed approximately 3 months after discharge.

**Conclusion:**

Discharge planning services for patients with diabetes during the hospital-to-home transition were commonly described as multicomponent interventions intended to support continuity of care. However, variations in delivery formats, outcome measures, follow-up periods, evaluation methods, and reporting quality highlight the need for further research with clearer intervention descriptions, consistent outcome measures, process evaluations, stakeholder perspectives, and longer-term outcome assessments. Future initiatives should prioritize continuous and adaptable discharge planning service models that are feasible, acceptable, and responsive to the needs of individuals with diabetes across different care contexts.

**Systematic Review Registration:**

https://archive.org/details/osf-registrations-3bnyq-v1, identifier doi: 10.17605/OSF.IO/3BNYQ.

## 1 Introduction

Diabetes mellitus is a chronic, progressive metabolic disorder characterized by persistent hyperglycemia resulting from impaired carbohydrate metabolism, reduced efficiency of glucose utilization, and excessive endogenous glucose production (1). According to the 11th edition of the international diabetes federation (IDF) Diabetes Atlas, the global number of adults living with diabetes was estimated at approximately 589 million in 2024 and is projected to rise to 853 million by 2050 (2). China continues to bear the largest national burden of diabetes worldwide, with the number of individuals living with diabetes expected to reach 168 million by 2050 (2). These projections suggest an accelerating global diabetes epidemic and highlight the critical importance of sustained self-management as a central component of comprehensive diabetes care (3).

In modern healthcare systems, efforts to optimize resource allocation and improve hospital bed turnover often necessitate patient discharge before complete clinical recovery (4). This challenge is particularly pronounced in contexts where healthcare resources remain unevenly distributed (5, 6). Despite the implementation of national strategies, such as the hierarchical medical system, to promote equitable access and rationalize healthcare delivery, primary healthcare institutions continue to face persistent constraints, including shortages of qualified personnel, limited medical equipment, and inadequate operational funding. Against this background, healthcare resources and services remain disproportionately concentrated in first-tier cities and tertiary hospitals (6). The post-discharge period is often characterized by inadequate utilization of available healthcare resources, which may contribute to fragmented follow-up care, insufficient patient education, and discontinuities in long-term disease management (6). In the context of early discharge, these gaps may increase the risk of adverse outcomes, including poor glycemic control, disease exacerbation, and unplanned hospital readmissions (7). Concurrently, economic pressures and the widespread adoption of “rapid recovery” pathways and early discharge models have accelerated the shift from hospital-based to home-based care. Although these approaches are intended to enhance system efficiency and promote timely recovery, they may also result in patients returning home before achieving sufficient readiness for independent self-management (8). Insufficient preparedness at discharge can compromise patients' ability to manage complex diabetes regimens during the transition period, thereby increasing the likelihood of disease relapse and unfavorable clinical outcomes (4, 8). Therefore, systematic assessment of discharge readiness, identification of post-discharge care needs, and the development of individualized, structured discharge plans have been increasingly emphasized as key elements of diabetes care transitions (4, 9).

Discharge planning represents a structured continuity-of-care model designed to support the transition from inpatient treatment to community- or home-based management (10, 11). This process typically begins at the time of hospital admission and involves comprehensive assessments of patients' physiological status, psychological well-being, social circumstances, and anticipated post-discharge care needs (9, 11–13). Based on these assessments, a multidisciplinary team, often comprising physicians, nurses, rehabilitation professionals, and social workers, collaboratively develops individualized discharge plans (12, 14). In this context, discharge planning is primarily a planning- and coordination-oriented, focusing on assessing needs, preparing patients and caregivers, and organizing post-discharge care to ensure continuity and readiness for transition (12, 15). Rather than being limited to a single intervention component, discharge planning represents a comprehensive and structured process that integrates assessment, education, coordination, and follow-up support. The primary objective of discharge planning is to ensure seamless continuity of medical and nursing care following hospital discharge, thereby enhancing patients' readiness for transition, supporting recovery, and promoting effective long-term self-management of chronic conditions (12, 16). Although discharge planning shares overlapping goals with transitional care, transitional care generally refers to a broader set of time-limited post-discharge care activities, which may include home visits, follow-up calls, and care coordination across settings (4). Evidence suggests that well-designed discharge planning interventions can shorten hospital stays, reduce the risk of readmission, and improve overall patient outcomes (16).

Although discharge planning is widely recognized as a critical component of care transitions in diabetes management, the existing literature remains fragmented. To date, few comprehensive syntheses have examined how discharge planning interventions for patients with diabetes are designed, delivered, and evaluated. In particular, it remains unclear whether and how these interventions provide continuous support as patients transition from the inpatient setting to the post-discharge phase. This issue is especially relevant for patients with diabetes, who are expected to manage medications, monitor blood glucose levels, modify lifestyle behaviors, and prevent complications after discharge. Accordingly, this scoping review aimed to map how discharge planning interventions for patients with diabetes are designed, delivered, and evaluated, and to identify gaps in intervention implementation, continuity of support, and outcome assessment. Guided by scoping review methodology (17), this review describes the key components of these interventions, with particular attention to nursing-led activities, delivery methods, intervention content, assessment time points, and outcome measures. By synthesizing evidence on the structure, delivery, and evaluation of discharge planning interventions, this review seeks to inform future research, clinical practice, and the development of more standardized discharge planning models or service systems for patients with diabetes.

## 2 Methods

This scoping review was conducted using the updated methodological framework developed by the Joanna Briggs Institute (JBI) Center for Evidence-Based Healthcare (17). The review process comprised several key stages: formulation of the research questions, development of comprehensive search strategies, establishment of inclusion and exclusion criteria, systematic extraction of relevant data, and synthesis of the findings. Accordingly, this review aimed to map the characteristics of existing intervention studies to inform the development of future intervention strategies. Accordingly, this review aimed to map implemented and empirically evaluated discharge planning interventions, including their components, delivery characteristics, assessment time points, and outcome measures. Although methodological quality appraisal is not mandatory for scoping reviews, we conducted a methodological quality appraisal to provide additional context for interpreting the included studies. The appraisal results were used descriptively and were not used to exclude studies or determine intervention effectiveness. The review was reported according to the Preferred Reporting Items for Systematic Reviews and Meta-Analyses extension for Scoping Reviews (PRISMA-ScR) guidelines (18). The study protocol was registered in the Open Science Framework (OSF) repository (doi: 10.17605/OSF.IO/3BNYQ).

### 2.1 Identifying the research questions

This scoping review sought to address the following research questions:

a) What types and components of interventions are incorporated into discharge planning for patients with diabetes?
b) What outcomes are reported in studies evaluating discharge planning interventions for patients with diabetes?

### 2.2 Data sources and search strategy

A systematic literature search was conducted across multiple electronic databases, including PubMed, Web of Science, Embase, CINAHL, CNKI, Wanfang, VIP, and SinoMed. The search strategy combined controlled vocabulary (e.g., subject headings) with free-text terms and covered publications from January 2020 to June 2025. This timeframe was chosen to capture recent discharge planning interventions for diabetes care and to minimize the inclusion of older studies that may not reflect current healthcare systems or contemporary discharge planning practices.

To maximize the retrieval of relevant studies, keywords related to diabetes and discharge planning were combined using Boolean operators (AND/OR). Diabetes-related terms included “diabetes mellitus,” “diabetic,” “diabetes,” “DM,” “impaired glucose tolerance,” and “glucose metabolism disorders.” Discharge-related terms included “patient discharge,” “discharge planning,” “discharge preparation services,” “discharge readiness,” “discharge preparation,” “transitional nursing,” and “continuing care.” The complete search strategies and corresponding retrieval results for each database are presented in Table 1.

**Table 1:** Search strategy.

Database	Search strategy	Results
PUBMED	#1 (“Diabetes Mellitus”[MeSH Terms] OR diabetes^*^[Title/Abstract] OR diabetic^*^[Title/Abstract] OR “DM”[Title/Abstract] OR “glycuresis”[Title/Abstract] OR “hyperglycemia”[Title/Abstract] OR “glucose intolerance”[Title/Abstract] OR “impaired glucose tolerance”[Title/Abstract] OR “glucose metabolism disorders”[Title/Abstract]) #2 (“Patient Discharge”[MeSH Terms] OR “discharge planning”[Title/Abstract] OR “discharge preparation services”[Title/Abstract] OR “discharge service”[Title/Abstract] OR “discharge readiness”[Title/Abstract] OR “discharge preparation”[Title/Abstract] OR “readiness for hospital discharge”[Title/Abstract] OR “discharge programme”[Title/Abstract] OR “discharge procedure”[Title/Abstract] OR “discharge guidance”[Title/Abstract] OR “discharge instruction”[Title/Abstract] OR “patient transfer”[Title/Abstract] OR “post discharge”[Title/Abstract] OR “hospital to home transition”[Title/Abstract] OR “transitional nursing”[Title/Abstract] OR “continuity of patient care”[Title/Abstract] OR “continuing care”[Title/Abstract] OR “care transition”[Title/Abstract]) #3 (“2020/01/01”[Date - Publication]: “2025/06/30”[Date - Publication]) #1 AND #2 AND #3	748
WEB OF SCIENCE	#1 TS=(“diabetes mellitus”) OR TS=(diabetes^*^) OR TS= (diabetic^*^) OR TS=(“DM”) OR TS=(“glycuresis”) OR TS= (“hyperglycemia”) OR TS=(“glucose intolerance”) OR TS=(“impaired glucose tolerance”) OR TS=(“glucose metabolism disorders”) #2 TS=(“patient discharge”) OR TS=(“discharge planning”) OR TS=(“discharge preparation services”) OR TS=(“discharge service”) OR TS=(“discharge readiness”) OR TS=(“discharge preparation”) OR TS=(“readiness for hospital discharge”) OR TS=(“discharge programme”) OR TS=(“discharge procedure”) OR TS=(“discharge guidance”) OR TS=(“discharge instruction”) OR TS=(“patient transfer”) OR TS=(“post discharge”) OR TS=(“hospital to home transition”) OR TS=(“transitional care”) OR TS=(“transitional nursing”) OR TS=(“care transition”) OR TS=(“continuity of patient care”) OR TS=(“continuing care”) #3 PY=(2020–2025) #1 AND #2 AND #3	121
EMBASE	#1 ‘diabetes mellitus'/exp OR ‘diabetes mellitus': ti, ab, kw OR diabetes: ti, ab, kw OR diabetic: ti, ab, kw OR ‘unspecified diabetes mellitus': ti, ab, kw #2 ‘hospital discharge'/exp OR ‘hospital discharge': ti, ab, kw OR ‘discharge planning': ti, ab, kw OR ‘discharge, hospital': ti, ab, kw OR ‘dismissal from hospital': ti, ab, kw OR ‘hospital dismissal':ti,ab,kw OR ‘patient discharge': ti, ab, kw OR ‘discharge preparation services': ti, ab, kw OR ‘discharge service': ti, ab, kw OR ‘discharge readiness': ti, ab, kw OR ‘discharge preparation': ti, ab, kw OR ‘readiness for hospital discharge': ti, ab, kw OR ‘discharge programme': ti, ab, kw OR ‘discharge procedure': ti, ab, kw OR ‘discharge guidance': ti, ab, kw OR ‘discharge instruction': ti, ab, kw OR ‘post discharge': ti, ab, kw OR ‘patient transfer': ti, ab, kw OR ‘hospital to home transition': ti, ab, kw OR ‘transitional care': ti, ab, kw OR ‘transitional nursing': ti, ab, kw OR ‘transition care': ti, ab, kw OR ‘care transition': ti, ab, kw OR ‘continuity of patient care': ti, ab, kw OR ‘continuing care': ti, ab, kw #3 [2020–2025]/py #1 AND #2 AND #3	244
CINAHL	#1 (MH “Diabetes Mellitus+” OR TI(diabetes^*^ OR diabetic^*^ OR DM OR glycuresis OR hyperglycemia OR “glucose intolerance” OR “impaired glucose tolerance” OR “glucose metabolism disorders”) OR AB(diabetes^*^ OR diabetic^*^ OR DM OR glycuresis OR hyperglycemia OR “glucose intolerance” OR “impaired glucose tolerance” OR “glucose metabolism disorders”)) #2 (MH “Patient Discharge+” OR TI(“patient discharge” OR “discharge planning” OR “discharge preparation service” OR “discharge service” OR “discharge readiness” OR “discharge preparation” OR “readiness for hospital discharge” OR “discharge programme” OR “discharge procedure” OR “discharge guidance” OR “discharge instruction” OR “transitional care” OR “transitional nursing” OR “patient transfer” OR “post discharge” OR “hospital to home transition” OR “continuity of patient care” OR “continuing care” OR “care transition” OR “transition care”) OR AB(“patient discharge” OR “discharge planning” OR “discharge preparation service” OR “discharge service” OR “discharge readiness” OR “discharge preparation” OR “readiness for hospital discharge” OR “discharge programme” OR “discharge procedure” OR “discharge guidance” OR “discharge instruction” OR “transitional care” OR “transitional nursing” OR “patient transfer” OR “post discharge” OR “hospital to home transition” OR “continuity of patient care” OR “continuing care” OR “care transition” OR “transition care”)) #3 PD 01/01/2020 - 06/30/2025 #1 AND #2 AND #3	456
CNKI	#1 SU=(Diabetes OR Hyperglycemia OR ‘Impaired Glucose Tolerance' OR ‘Glucose Metabolism Disorders') #2 SU=(‘Discharge Planning' OR ‘Discharge Preparation Services' OR ‘Discharge Preparation' OR ‘Transitional Care' OR ‘Continuing Care') #3 PT=2020–2025 #1 AND #2 AND #3	461
		
Wanfang	#1 Subject: (Diabetes OR Hyperglycemia OR Impaired Glucose Tolerance OR Abnormal Glucose Metabolism) #2 Subject: (Discharge Preparation Services OR Discharge Planning OR Discharge Preparation OR Transitional Care OR Continuity of Care) #3 Year:2020–2025 #1 AND #2 AND #3	827
VIP	#1 M=(Diabetes OR Hyperglycemia OR Impaired Glucose Tolerance OR Abnormal Glucose Metabolism) #2 M=(Discharge Planning OR Discharge Preparation Services OR Discharge Preparation OR Transitional Care OR Continuing Care) #3 Y=2020–2025 #1 AND #2 AND #3	1,100
SinoMed	#1 (“Diabetes” [Common field: Smart] OR “Hyperglycemia” [Common field: Smart] OR “Impaired glucose tolerance” [Common field: Smart] OR “Abnormal glucose metabolism” [Common field: Smart]) #2 (“Discharge Preparation Services” [Common field: Smart] OR “Discharge Planning” [Common field: Smart] OR “Discharge Preparation” [Common field: Smart] OR “Continuing Care” [Common field: Smart] OR “Transitional Care” [Common field: Smart]) #3 PY=2020–2025 #1 AND #2 AND #3	513

### 2.3 Inclusion and exclusion criteria

#### 2.3.1 Inclusion criteria

Study eligibility was determined using the Population-Concept-Context (PCC) framework recommended by the Joanna Briggs Institute (JBI) scoping review methodology (17).

(1) Participants (P): The population of interest comprised adults diagnosed with type 1 diabetes, type 2 diabetes, or gestational diabetes adults aged 18 years or older.
(2) Concept (C): The concept focused on discharge planning and related interventions specifically designed for these patients.
(3) Context (C): The context encompassed healthcare settings in which patients received discharge planning interventions, with sufficient detail provided regarding the content and delivery of the interventions.
(4) Study type: Eligible studies were randomized controlled trials (RCTs) and quasi-experimental studies. This restriction was applied because the review focused on implemented and evaluated discharge planning interventions, including their components, delivery characteristics, outcomes, and evaluation approaches. Studies using other empirical designs were outside the scope of this review.

#### 2.3.2 Exclusion criteria

Studies were excluded if they met any of the following criteria:

(1) Studies that did not focus on discharge planning services during the hospital-to-home transition.
(2) Studies that lacked a detailed description of the intervention content.
(3) Duplicate publications.
(4) Studies for which the full text was unavailable.
(5) Conference abstracts, narrative reviews, systematic reviews, clinical practice guidelines, and other forms of non-original research.
(6) Publications not written in Chinese or English.

### 2.4 Data extraction and quality assessment

All retrieved records were imported into Zotero reference management software, and duplicate entries were removed. Two reviewers independently screened titles and abstracts and subsequently assessed full texts for eligibility. Articles with unavailable full texts or content unrelated to the review objectives were excluded. Discrepancies at any stage of the screening process were resolved through discussion with a third reviewer. When necessary, language-related uncertainties were clarified through consultation with a native English-speaking professor. Data extraction was independently performed by two reviewers, and disagreements were resolved through consultation with a third reviewer or group discussion, as needed. Data were extracted from all included studies using a standardized extraction form. Extracted information included authors, year of publication, country, study design, sample size, disease type, intervention or program description, measurement time points, and reported outcome measures. Key intervention components, delivery methods, and primary objectives were further summarized to provide a detailed overview of how discharge planning interventions were structured and delivered across the included studies.

Methodological quality was assessed using design-specific appraisal tools. Randomized controlled trials were evaluated using the revised Cochrane Risk of Bias 2 tool for randomized trials (19), whereas quasi-experimental studies were assessed using the revised JBI Critical Appraisal Checklist for Quasi-Experimental Studies (20). Two reviewers independently appraised all included studies, and any disagreements were resolved through discussion, with consultation from a third reviewer when necessary.

## 3 Results

### 3.1 Literature search results

**Figure 1:**
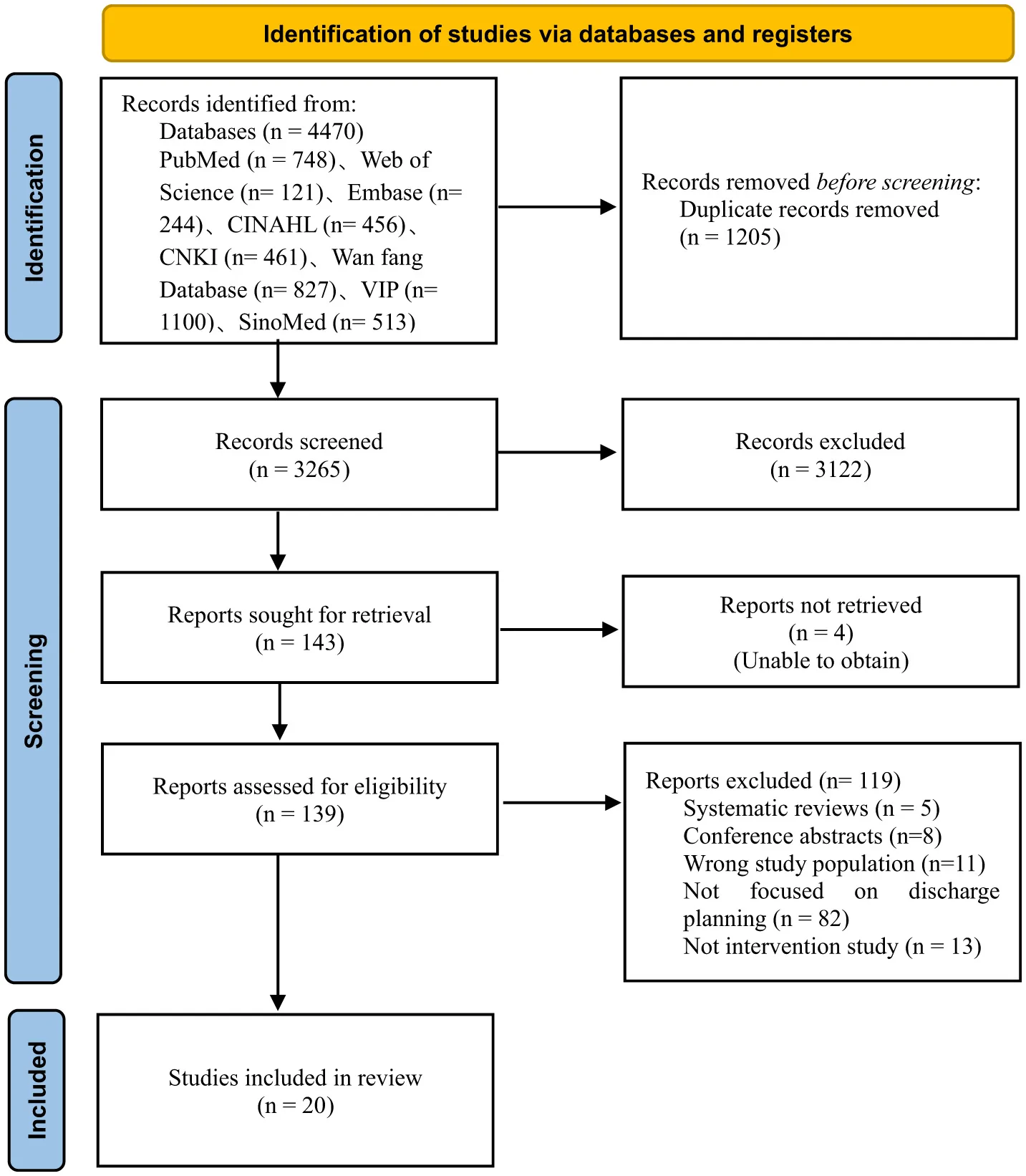
The flow diagram for the identification, screening, and eligibility of studies (PRISMA-ScR).

A total of 4,470 records, including 1,568 English-language publications and 2,902 Chinese-language publications, were initially retrieved from the selected databases and imported into Zotero for management. After removal of 1,205 duplicate records, titles and abstracts of 3,265 records were screened, yielding 143 potentially relevant studies. Four studies were excluded because the full texts could not be obtained, and the remaining 139 full-text articles were assessed for eligibility. Full-text screening resulted in the exclusion of 119 studies for the following reasons: systematic reviews (*n* = 5), conference abstracts (*n* = 8), ineligible study populations (*n* = 11), lack of focus on discharge planning interventions (*n* = 82), and non-intervention studies (*n* = 13). Ultimately, 20 studies were included in this scoping review, of which 14 were published in Chinese-language journals and 6 in English-language journals. The study selection process is summarized in the PRISMA-ScR flow diagram (Figure 1) (18).

### 3.2 General characteristics of the selected studies

Table 2 summarizes the main characteristics of the included studies. Among the 20 included studies, 17 were conducted in China and three in the United States. Sixteen studies used randomized controlled trial designs, whereas four used quasi-experimental designs. All studies were published between 2020 and 2025.

**Table 2:** Characteristic of the included articles.

Author	Year	Country	Study design (RCT/non-RCT)	Sample size (test group/control group)/case	Disease type	Intervention/program	Measurement time points	Outcome measures
Wang et al. (21)	2025	China	Non-RCT	100 (50/50)	Type 2 diabetes	Timing Theory-Based Discharge Preparation Service	Baseline, Day 7 post-intervention, Month 1 post-discharge, Month 3 post-discharge	1.Readiness for discharge 2.Quality of discharge instructions 3.Self-management level
Bai. (32)	2025	China	Non-RCT	80 (39/41)	Type 2 diabetes	Hospital-to-Home Transitional Care Plan	Baseline, Post-intervention	1.FPG, 2hPG 2.Self-management level 3.Quality of life 4.Unplanned readmission rate
Lu et al. (33)	2024	China	RCT	130 (65/65)	Mixed (T1DM and T2DM)	Admission-to-Home Health Education Program	Baseline, Post-intervention	1.FPG, 2hPG, HbA1c 2.Insulin Injection adherence 3.Mastery of standardized insulin injection 4.Quality of life
Wang et al. (26)	2024	China	RCT	140 (66/74)	Type 2 diabetes	Shared-Care Self-Management Plan	Baseline, Day 3 of hospitalization, At discharge, Post-intervention	1.HbA1C, FPG, 2hPG 2.Self-management level 3.Readiness for discharge
Si et al. (27)	2024	China	Non-RCT	86	Type 2 diabetes	MMC-Based Continuing Care Plan	Baseline, Month 1 post-intervention, Month 3 post-intervention	1.FPG, 2hPG, BMI 2.Self-management level 3.Quality of life
Huang (22)	2024	China	RCT	100 (50/50)	Type 2 diabetes	Stages of Change Theory-Based Discharge Preparation Program	Baseline, Month 3 post-intervention	1.FPG, 2hPG, HbA1C 2.Readiness for discharge 3.Self-management level
Guo (28)	2024	China	Non-RCT	184 (92/92)	Mixed (T1DM and T2DM)	Mobile Application-Based Hospital–Home Collaboration Program	Baseline, Month 3 post-intervention	1.FPG, 2hPG, HbA1C, BMI 2.Self-management level 3.Quality of life
Cheng (34)	2023	China	RCT	66 (33/33)	Type 2 diabetes	Discharge Preparation Nursing Service	Baseline, Post-intervention	FPG, 2hPG, HbA1C
Chen (29)	2023	China	RCT	100 (50/50)	Mixed (T1DM and T2DM)	Diabetes Specialist Nurse-Led MMC-Based Hospital-to-Home Care Program	Baseline, Post-intervention	1.FPG, 2hPG, HbA1C 2.Self-management level 3.Quality of life 4.Nursing satisfaction
White et al. (35)	2022	America	RCT	158 (82/76)	Type 2 diabetes	Diabetes-Focused Electronic Discharge Order Set	At discharge, Week 12 post-discharge, Week 24 post-discharge	1.HbA1C, FPG 2.Proportion of patients with prescriptions for insulin and related supplies; proportion receiving clear discharge instructions
Rubin et al. (36)	2022	America	RCT	91 (45/41)	Mixed (T1DM and T2DM)	Diabetes Transition of Hospital Care Program (DiaTOHC)	Baseline, Post-intervention	1.All-cause unplanned hospital readmission within 30 days of discharge 2.Rate of ED visit not associated with hospital admission 3.Composite rate of unplanned readmission 4.Cost of post-discharge acute care and intervention 5.Daily frequency of SMBG testing 6.Hypoglycemia incidence
Guo et al. (23)	2022	China	RCT	50 (25/25)	Type 2 diabetes	Interactive Goal Attainment Theory-Based WeChat Continuing Care Program	Baseline, Post-intervention, Month 6 post-intervention	1.FPG, 2hPG 2.Anxiety level 3.Medication adherence 4.Diet and exercise adherence 5.Quality of life
Lyu et al. (37)	2021	China	RCT	106 (54/52)	Type 2 diabetes	Nurse-Led Web-Based Transitional Care Program	Baseline, Month 3 post-intervention	1.HbA1C 2.Treatment adherence 3.Self-efficacy 4.Quality of life
Cheng et al. (38)	2021	China	RCT	242 (121/121)	Type 2 diabetes	Empowerment-Based Self-Management Program	Baseline, Week 1 post-intervention, Month3 post-intervention	1.Empowerment level 2.Diabetes distress 3.Quality of life
Magny-Normilus et al. (39)	2021	America	RCT	180 (88/92)	Type 2 diabetes	Multidisciplinary Intensive Discharge Intervention	Baseline, Post-intervention	1.Insulin injection adherence within 90 days post-discharge 2.HbA1C 3.Incidence of hypoglycemia within 30 days post-discharge 3.Readmission rate within 30days post-discharge
Liu et al. (40)	2021	China	RCT	209 (105/104)	Type 2 diabetes	Internet-Based Continuing Care Program	Baseline, Post-intervention	1. FPG, 2hPG, HbA1C, TG, TC, LDL-C, HDL-C, BMI 2.Self-management level 3.Incidence of mood disorders
Ge et al. (24)	2021	China	RCT	140 (70/70)	Type 2 diabetes	ITHBC-Based WeChat Continuing Care Program	Baseline, Month 3 post-intervention, Month 6 post-intervention	1. FPG, HbA1C, TC, TG, LDL-C, HDL-C, ALT, AST, BUN, Cr 2.Self-management level 3.Qualityof life
Zhao et al. (30)	2020	China	RCT	220 (110/110)	Type 2 diabetes	MMC-Based Standardized Continuing Care Management Model	Baseline, Post-intervention	1. FPG, 2hPG, HbA1C 2.Self-management level 3.Nursing satisfaction
Shi (31)	2020	China	RCT	95 (48/47)	Type 2 diabetes	Multidisciplinary Discharge Planning Service	Baseline, Post-intervention	1.Readiness for discharge 2.Self-efficacy
Fan (25)	2020	China	RCT	200 (100/100)	Type 2 diabetes	Time-Efficacy Theory-Based Continuing Care Program	Baseline, Month 6 post-intervention, Month 12 post-intervention	1. FPG, 2hPG 2. Self-care ability 3. Quality of life

### 3.3 Risk of bias in studies

Risk of bias in the 16 randomized controlled trials was assessed using the Cochrane Risk of Bias 2 (RoB 2) tool. Two studies were judged to have a low overall risk of bias, whereas the remaining 14 were judged to have some concerns, as shown in Table 3. These concerns mainly arose from insufficient or unclear reporting of the randomization process, allocation concealment, blinding procedures, handling of missing outcome data, outcome measurement, and selection of the reported results. The four quasi-experimental studies were assessed using the JBI Critical Appraisal Checklist for Quasi-Experimental Studies, with the results presented in Table 4. Only one study met all appraisal criteria. The remaining studies showed methodological limitations or insufficient reporting, mainly related to temporal precedence, selection and allocation procedures, administration of the intervention or exposure, outcome assessment and measurement, and participant retention.

**Table 3:** Risk of bias assessment of the included RCTs (*n* = 16).

Reference	Randomization process	Deviations from the intended intervention	Missing outcome data	Measurement of the outcome	Selection of the reported result	Overall risk
Lu et al. (33)	Low	Some concerns	Some concerns	Low	Some concerns	Some concerns
Wang et al. (26)	Low	Some concerns	Low	Some concerns	Some concerns	Some concerns
Huang (22)	Low	Some concerns	Low	Some concerns	Some concerns	Some concerns
Cheng (34)	Low	Low	Some concerns	Low	Some concerns	Some concerns
Chen (29)	Low	Some concerns	Some concerns	Low	Some concerns	Some concerns
White et al. (35)	Low	Low	Low	Low	Low	Low
Rubin et al. (36)	Low	Low	Low	Low	Low	Low
Guo et al. (23)	Low	Some concerns	Some concerns	Some concerns	Some concerns	Some concerns
Lyu et al. (37)	Some concerns	Low	Low	Low	Low	Some concerns
Cheng et al. (38)	Low	Low	Some concerns	Some concerns	Low	Some concerns
Magny-Normilus et al. (39)	Low	Low	Low	Low	Some concerns	Some concerns
Liu et al. (40)	Low	Low	Some concerns	Low	Some concerns	Some concerns
Ge et al. (24)	Low	Low	Some concerns	Low	Some concerns	Some concerns
Zhao et al. (30)	Low	Some concerns	Some concerns	Low	Some concerns	Some concerns
Shi (31)	Some concerns	Low	Some concerns	Some concerns	Some concerns	Some concerns
Fan (25)	Low	Low	Some concerns	Some concerns	Some concerns	Some concerns

**Table 4:** Risk of bias assessment of the included quasi-experimental studies (*n* = 4).

Reference	Temporal precedence	Selection and allocation	Confounding actors	Administration of intervention/exposure	Assessment, detection, and measurement of the outcome			Participant retention	Statistical conclusion validity
	Q1	Q2	Q3	Q4	Q5	Q6	Q7	Q8	Q9
Wang et al. (21)	UC	Y	Y	UC	Y	UC	Y	UC	Y
Bai (32)	UC	Y	Y	Y	N	UC	Y	N	UC
Si et al. (27)	Y	N	N/A	N/A	Y	Y	Y	UC	Y
Guo (28)	Y	Y	Y	Y	Y	Y	Y	Y	Y

### 3.4 Characteristics and components of diabetes discharge planning interventions

Table 5 provides a detailed overview of the characteristics of discharge planning interventions. The following subsections describe their theoretical frameworks, delivery methods, and intervention components.

**Table 5:** Intervention components, objectives, and delivery methods of the included studies.

Author	Intervention components	Delivery method	Primary objectives
Wang et al. (21)	(1) Comprehensive assessment within 24 h of admission. (2) Staged education across disease onset, stabilization, and pre-discharge phases. (3) Collaborative goal-setting with patients and families. (4) WeChat-based follow-up at 7 days, 1 month, and 3 months with individualized plan adjustment.	Face-to-face	Improve discharge readiness
Bai. (32)	(1) Pre-discharge assessment of care needs and transition barriers. (2) Intensive inpatient and early post-discharge education (30–60 min, 4 times/week). (3) Family-based emotional support (4) WeChat-based monitoring and treatment adjustment at weeks 1–4.	Face-to-face and remote delivery via WeChat	Improve glycemic control
Lu et al. (33)	(1) Biweekly structured inpatient education. (2) Individualized instruction and skills demonstration. (3) Discharge logbooks and WeChat reminders. (4) Monthly home visits over 6 months addressing insulin use, diet, exercise, and psychosocial health.	Face-to-face and remote delivery via WeChat	Improve glycemic control
Wang et al. (26)	(1) Electronic health record establishment. (2) Mobile app-assisted self-management (3) Staged inpatient education (diet, exercise, glucose monitoring). (4) Discharge readiness assessment. (5) Follow-up at 1 and 6 months.	Remote delivery via the Yutang app	Improve discharge readiness
Si et al. (27)	(1) Integrated in-hospital and out-of-hospital data via MMC platform. (2) Automated data analysis and early warnings. (3) Scheduled follow-ups at 1–12 weeks. (4) Periodic health education lectures.	Remote delivery via the MMC platform	Enhance self-management ability
Huang. (22)	(1) Stage-specific education and behavioral training from admission to post-discharge. (2) Readiness assessment. (3) WeChat/telephone follow-ups at weeks 4 and 8. (4) Outpatient evaluation after week 12.	Face-to-face and remote delivery via WeChat	Improve discharge readiness
Guo. (28)	(1) Individualized discharge planning. (2) App-based reminders, education pushes, and monitoring. (3) Frequent early follow-ups transitioning to monthly contacts. (4) Quarterly outpatient visits.	Remote delivery via a diabetes app	Improve glycemic control
Cheng. (34)	(1) In-hospital assessment and individualized education (2) Post-discharge multidisciplinary follow-ups via WeChat, telephone, or home visits at defined intervals.	Face-to-face and remote delivery via telephone	Improve glycemic control
Chen. (29)	(1) Multidisciplinary MMC team. (2) App-based patient data uploads and automated plan adjustment. (3) Multimodal inpatient education. (4) Standardized post-discharge management protocols.	Face-to-face and remote delivery via the MMC platform	Improve glycemic control
White et al. (35)	(1) Electronic discharge order templates with decision support. (2) Telephone follow-ups at 2 and 6 weeks focusing on glucose monitoring, insulin use, and appointment reminders.	Remote delivery and remote delivery via telephone	Improve glycemic control
Rubin et al. (36)	(1) Individualized discharge education aligned with ADA guidelines. (2) HbA1c-guided treatment adjustment. (3) Early post-discharge telephone support.	Face-to-face and remote delivery via telephone	Reduce 30-day unplanned hospital readmissions
Guo et al. (23)	(1) Individualized continuity-of-care planning. (2) WeChat group engagement. (3) Weekly education and biweekly follow-ups. (4) Monthly goal evaluation and psychological support.	Face-to-face and remote delivery via WeChat	Reduce anxiety and depressive symptoms
Lyu et al. (37)	(1) Personalized self-management plans uploaded online. (2) Structured online courses. (3) Peer interaction. (4) 24/7 nurse consultation. (5) Remote data collection.	Face-to-face and remote delivery via online courses	Improve glycemic control
Cheng et al. (38)	(1) Individual motivational communication. (2) Group education sessions. (3) Personalized telephone coaching focused on SMART goals and daily integration.	Face-to-face and remote delivery via telephone	Reduce anxiety and depressive symptoms
Magny-Normilus et al. (39)	(1) Standardized post-discharge insulin regimen. (2) Structured discharge education. (3) Early telephone follow-up. (4) Home visits. (5) Remote glucose monitoring. (6) Timely clinic review.	Face-to-face and remote delivery via telephone	Improve diabetes treatment adherence
Liu et al. (40)	(1) App and WeChat onboarding during hospitalization. (2) Individualized follow-up planning. (3) Daily glucose review. (4) Physician-led treatment adjustment. (5) Weekly online education.	Remote delivery via the hospital's official app	Improve glycemic control
Ge et al. (24)	(1) Systematic assessment. (2) WeChat-based guidance. (3) Ongoing online social support	Face-to-face and remote delivery via WeChat	Improve glycemic control
Zhao et al. (30)	(1) Comprehensive patient records. (2) Regular telephone monitoring. (3) Individualized nurse-led education. (4) Group lectures. (5) Psychosocial support.	Face-to-face and remote delivery via the MMC platform	Improve glycemic control
Shi. (31)	(1) Comprehensive assessment. (2) Structured discharge counseling. (3) Dynamic post-discharge follow-up via telephone or WeChat.	Face-to-face and remote delivery via public account push notifications	Improve discharge readiness
Fan. (25)	(1) Self-care handbook distribution. (2) Standardized health records. (3) WeChat education platform. (4) Emotional, needs-based, and role-model incentives.	Face-to-face and remote delivery via WeChat	Enhance self-management ability

#### 3.4.1 Theoretical frameworks underpinning discharge planning interventions

Five studies (21–25) explicitly reported that their discharge planning interventions were informed by established theoretical frameworks. These frameworks included Timing Theory (21), the Stages of Behavioral Change Theory (22), Goal Attainment Theory (23), Health Behavior Change Integration Theory (24), and Timeliness Incentive Theory (25).

#### 3.4.2 Delivery methods of discharge planning interventions

Remote delivery approaches included mobile health application-based interventions (*n* = 5) (26–30) and multichannel digital interventions incorporating social media and telehealth components (*n* = 18) (21–25, 28–40). Because some studies incorporated multiple delivery approaches, the categories were not mutually exclusive. Mobile health application-based interventions commonly utilized the National Metabolic Management Center (MMC) platform (27, 29, 30) and other mobile health applications (26, 28) to provide diabetes education, health monitoring, and post-discharge follow-up support. Multichannel digital interventions involved multiple delivery elements, and some studies used more than one component. These included social media-based approaches, such as WeChat groups (21–25, 30, 32–34, 40), and public account push notifications (24, 25, 31, 40), as well as other telehealth delivery methods, such as telephone follow-ups (21, 22, 28, 30, 31, 34–36, 38, 39) and online courses (37), to enhance patient engagement and continuity of care.

Face-to-face interventions comprised both group-based (*n* = 7) (22, 25, 27, 29, 33, 37, 38) and individual-based formats (*n* = 17) (21, 23, 25–32, 34–40). These interventions typically involved structured health education sessions, individualized counseling, interactive question-and-answer sessions, group discussions, and behavior change counseling.

#### 3.4.3 Intervention content

Multidisciplinary comprehensive assessment was reported in eight studies (21–23, 26, 28, 30–32). Discharge planning was typically delivered by multidisciplinary teams comprising endocrinologists, diabetes specialist nurses, dietitians, and rehabilitation therapists, with the involvement of patients and their caregivers. Comprehensive assessments encompassed patients' physical and psychological status, self-management capacity, disease-related knowledge, treatment adherence, anticipated post-discharge needs, potential transition barriers, and available social support. Assessments were conducted at different time points across studies, including within 24 h of admission, within 3 days of admission, 24 h before discharge, and 3 days before discharge. Assessment modalities mainly included face-to-face evaluations and digital assessments conducted through the National Metabolic Management Center (MMC) platform.

Patient education was reported in all 20 studies (21–40). Educational interventions focused on core diabetes-related knowledge, including disease mechanisms, treatment modalities, the importance of self-management, and awareness of diabetes-related risks and complications. Education was commonly delivered through printed diabetes management manuals, instructional videos, health education materials, and brief structured education sessions. For example, Bai (32) implemented structured diabetes education courses, whereas Huang (22) facilitated patient self-learning through standardized diabetes management manuals.

Self-management support was included in all 20 studies (21–40) and represented a core component of discharge planning interventions. As an overarching intervention component, self-management support encompassed several practical and behavioral elements related to post-discharge diabetes management, including dietary self-management, exercise guidance, medication management, blood glucose monitoring, insulin injection training, complication prevention, and psychological support. Dietary self-management was the most commonly reported element and was included in all studies (21–40). It addressed food exchange, portion control, meal planning, cooking methods, and dietary precautions related to diabetes-associated complications. Exercise guidance was reported in 15 studies (22, 23, 25–34, 36, 37, 39) and included recommendations on the benefits, types, intensity, and safety precautions of physical activity. Patients were commonly advised to engage in regular, moderate exercise and to avoid excessive or strenuous activity that could contribute to glycemic instability. Some studies also addressed the management of exercise-related emergencies. Medication management was included as another element of self-management support in 12 studies (22, 25, 26, 28–30, 32, 34–37, 39), covering the classification and appropriate use of hypoglycemic agents, administration timing and frequency, potential adverse effects, and adherence to prescribed treatment regimens. Blood glucose monitoring guidance was reported in 11 studies (22, 26, 28, 29, 32–35, 37, 38, 40). This element focused on the significance of self-monitoring, target glucose ranges, appropriate monitoring time points, and correct testing techniques. Insulin injection training was included in eight studies (21, 22, 26, 28, 32, 33, 39, 40) and covered insulin types, injection-site selection and rotation, dosing accuracy, injection timing, insulin storage, and injection-related precautions. For instance, Lu et al. (33) developed a standardized insulin injection manual and assessed insulin adherence as an outcome. Complication prevention guidance was reported in four studies (25, 26, 32, 37), including monitoring foot health, blood pressure, lipid levels, and visual function, as well as recognizing and responding to symptoms of hyperglycemia and hypoglycemia. In addition to behavioral and technical self-management skills, psychological support was incorporated in seven studies (21, 23, 25, 26, 28, 30, 32). This component included tailored emotional counseling, motivational strategies, educational videos documenting patients' diabetes management experiences, and role model-based encouragement. These strategies addressed emotional distress, coping, self-confidence, motivation, and engagement in diabetes self-management.

Follow-up strategies were reported in 16 studies (21–25, 27–34, 36, 39, 40) and represented an important component of post-discharge support. These strategies involved structured contact with patients after discharge to monitor diabetes management, identify self-management difficulties, answer questions, and provide individualized guidance. Follow-up content commonly addressed blood glucose monitoring, medication adherence, diet and exercise management, insulin use, symptom recognition, complication prevention, and appointment scheduling. Follow-up was commonly delivered through telephone consultations, WeChat-based communication, online platforms, and home visits. The timing and frequency of follow-up varied across studies, with common follow-up points including 1 week, 1 month, and 3 months after discharge.

### 3.5 Outcome measures and reported outcomes of diabetes discharge planning

#### 3.5.1 Clinical outcomes

Seventeen studies (22–30, 32–37, 39, 40) reported clinical outcomes related to post-discharge glycemic control and metabolic status following discharge planning interventions. The most frequently reported indicators included fasting plasma glucose (FPG), two-hour postprandial glucose (2hPG), glycated hemoglobin (HbA1c), and body mass index (BMI). These measures were used to describe changes in glycemic control following discharge planning interventions. Most studies observed reductions in FPG, 2hPG, HbA1c, or BMI. Two studies (35, 39) also reported a lower incidence of hypoglycemic events after the intervention period.

#### 3.5.2 Self-management and behavioral outcomes

Eighteen studies (21–33, 35–37, 39, 40) evaluated self-management and behavioral outcomes, including self-management capacity, self-efficacy, discharge readiness, quality of discharge education, and adherence-related behaviors. Self-management capacity was commonly assessed using standardized instruments, including the Diabetes Self-Management Behavior Scale (21, 24, 26, 28–30, 32, 40), Diabetes Self-Management Scale (22), Diabetes Self-Care Scale (27), and Self-Care Competence Scale (25). Self-efficacy was measured using the Chronic Disease Self-Efficacy Scale (37) and the General Self-Efficacy Scale (31). Discharge readiness and the quality of discharge instructions were assessed using the Discharge Readiness Scale (21, 22, 26, 31) and the Discharge Instruction Quality Scale (21), respectively.

Across the included studies, discharge readiness, self-management capacity, and adherence-related outcomes were reported as outcome measures in discharge planning interventions for patients with diabetes. One study (33) reported changes in patients' knowledge, attitudes, and behaviors related to insulin injection following a structured discharge readiness program. Another study (36) reported increased adherence to regular blood glucose monitoring after a discharge planning intervention. Adherence-related outcomes included overall treatment adherence (37), medication adherence (23, 35), dietary and exercise adherence (23), and adherence to insulin injection regimens (33, 39). Collectively, these outcomes reflected a focus on patients' readiness and capacity to engage in diabetes-related self-management after discharge.

#### 3.5.3 Healthcare utilization outcomes

Three studies (32, 36, 39) examined healthcare utilization and related cost outcomes, with a primary focus on unplanned hospital readmissions after discharge. These outcomes reflected post-discharge healthcare service use and care-related costs. One study (36) reported that patients receiving a hospital-based diabetes care transition program had lower unplanned readmission rates and overall treatment costs. The other two studies (32, 39) assessed unplanned readmission-related indicators but did not observe statistically significant differences between intervention and control groups. Overall, healthcare utilization outcomes were reported in fewer studies than clinical and self-management outcomes.

#### 3.5.4 Patient-reported outcomes

Ten studies (23–25, 27–29, 32, 33, 37, 38) evaluated patient-reported outcomes, including quality of life, patient empowerment, diabetes-related distress, anxiety, and patient satisfaction. Patient-reported measures provided insight into patients' perceived health status, emotional wellbeing, confidence in diabetes management, and satisfaction with discharge-related care. Across these studies, reported findings were generally favorable for the patient-reported outcomes assessed, including quality of life and patient empowerment (38), diabetes-related distress and anxiety (23, 38), and satisfaction with discharge-related care (27). The specific patient-reported outcomes assessed varied across studies.

## 4 Discussion

This scoping review provides a comprehensive synthesis of existing research on discharge planning for patients with diabetes, encompassing delivery formats, nursing roles, intervention components, implementation strategies, and reported outcomes. The findings indicate that discharge planning interventions were commonly delivered through face-to-face, multichannel digital, and follow-up-based approaches. These interventions frequently incorporated diabetes education, lifestyle counseling, self-management support, and follow-up strategies, highlighting the multidimensional nature of discharge planning for patients with diabetes. The outcomes reported across the included studies mainly covered glycemic indicators, self-management behaviors, treatment adherence, psychological outcomes, quality of life, and patient satisfaction.

The delivery formats of discharge planning reflect the growing integration of digital technologies into transitional care for patients with diabetes. Web-based and mobile health interventions have become increasingly prominent, in supporting diabetes self-management support, health monitoring, and post-discharge follow-up (41). Advances in technology and intelligent interaction systems have contributed to the development of mHealth (41, 42), with applications incorporating functions related to treatment adherence, clinical indicator monitoring, and long-term care management (41). In addition, mobile health platforms are consistent with the “Internet Plus Nursing” initiative, as they enable patients to access healthcare services without requiring in-person visits to hospitals or community health centers (42). This mode of service delivery may be especially relevant in regions with limited healthcare resources. The diversity of delivery formats identified in this review also suggests that discharge planning interventions are influenced by local healthcare resources, digital infrastructure, and patient communication preferences. For example, several studies used China-specific digital platforms, such as WeChat and the National Metabolic Management Center (MMC), or group-based formats, reflecting adaptations to specific care contexts.

The findings also suggest that discharge planning for patients with diabetes is commonly conceptualized as a multidisciplinary and multicomponent care process. The involvement of endocrinologists, diabetes specialist nurses, dietitians, rehabilitation therapists, pharmacists, and psychologists reflects the complex care needs of patients during the transition from hospital to home (16, 43). Across the included studies, discharge planning typically included patient assessment, structured education, lifestyle counseling, blood glucose monitoring, psychological support, and post-discharge follow-up. This pattern suggests that discharge planning for patients with diabetes extends beyond pre-discharge education and preparation to include ongoing post-discharge support. In addition, some interventions used established theoretical frameworks to guide behavior change and self-management support. The outcome domains reported in these studies included glycemic indicators, discharge readiness, quality of discharge education, health behaviors, and self-management-related outcomes (21–25). This range of outcomes reflects the potential role of discharge planning in supporting both short-term clinical stability and longer-term diabetes self-management after discharge. In this regard, health education and self-management support remain central components to this process (44, 45), as patients need to apply a range of self-management knowledge and skills in their daily routines, including lifestyle modification, medication and insulin management, blood glucose monitoring, and complication prevention (46, 47).

### 4.1 Gaps in literature and future research challenges

Discharge planning has been increasingly investigated in diabetes care; however, several gaps remain in the current literature. One notable gap concerns study design and generalizability. Many of the included studies had small sample sizes and used single-center designs, which may limit the generalizability of findings across different healthcare settings, geographic regions, and patient populations. Future research should therefore consider large-scale, multicenter studies to further examine the implementation, feasibility, and applicability of discharge planning models for patients with diabetes.

Methodological rigor and reporting transparency also remain important concerns. Although this scoping review primarily aimed to map the existing evidence rather than determine intervention effectiveness, the risk-of-bias and critical appraisal findings indicate that many included studies had limitations in methodological conduct, intervention reporting, outcome assessment, and follow-up procedures. These limitations may influence the interpretation of the available evidence. In addition, beyond methodological quality, limited reporting of process evaluation made it difficult to determine whether interventions were delivered as planned, completed by participants, and implemented in routine clinical practice. Future studies should use more rigorous and transparently reported designs, provide clearer descriptions of methodological procedures, and strengthen the reporting of intervention components, implementation processes, and follow-up procedures.

Caregiver involvement represents another underdeveloped area. Although some studies mentioned caregiver engagement, caregiver-related intervention components were often insufficiently described, and caregivers were not fully integrated into the discharge planning process. Given the important role that caregivers may play in supporting diabetes self-management during the transition from hospital to home, future interventions should explicitly consider caregivers' needs and roles. Discharge planning approaches involving both patients and caregivers may help support discharge readiness and long-term self-management after discharge.

Variation in intervention timing and follow-up duration was also evident throughout the included studies, which may have limited the comparability of findings. Discharge planning should be understood as a continuous process that begins during hospitalization and extends into the post-discharge period to support continuity of care across the hospital-to-home transition. However, this continuity was not clearly reflected in some included studies. In these studies, interventions tended to focus mainly on the pre-discharge or early post-discharge phases, and follow-up was commonly limited to 1–3 months after discharge. Consequently, evidence on the longer-term outcomes of discharge planning interventions remains limited. Future studies should clearly define intervention timing, duration, intensity, and follow-up procedures, extend follow-up periods, and systematically assess long-term outcomes.

Outcome selection and measurement also require further attention. The included studies commonly assessed outcomes such as glycemic control, self-management capacity, and quality of life. However, outcome measures and assessment approaches were not consistently applied across studies, which may limit direct comparison of reported outcomes. Only a small number of studies evaluated adverse clinical outcomes, such as hypoglycemia. In addition, assessments of self-management, discharge readiness, and quality of life were mainly based on patient-reported measures, with limited input from other stakeholders, such as caregivers and healthcare professionals. Future studies should consider greater consistency in outcome selection and measurement, while also broadening outcome domains to include adverse events and perspectives from multiple stakeholders.

### 4.2 Implications for research

This scoping review mapped the existing literature on the role of discharge planning services in supporting continuity of care for patients with diabetes during the hospital-to-home transition. Future research should not only assess the effectiveness of discharge planning interventions but also examine how these interventions are delivered in routine practice and which factors influence their implementation. This is particularly important because discharge planning is a complex, multicomponent process that may be shaped by healthcare resources, discharge procedures, continuity-of-care structures, and patient-provider interactions.

As the included studies were predominantly conducted in China, further research is needed to examine whether the current findings are applicable to other healthcare systems. Contextual factors, including family involvement in post-discharge care, patients' expectations regarding professional guidance, and patterns of healthcare utilization, may influence diabetes self-management after discharge and affect the design and delivery of discharge planning interventions. Future studies should therefore examine discharge planning models across different healthcare systems and sociocultural contexts. Well-designed and clearly reported studies would help determine which components of discharge planning can be applied in different healthcare settings and which require adaptation for local practice.

### 4.3 Limitations

This review has several limitations. First, variations in study design, intervention components, follow-up duration, and outcome measurement across the included studies should be considered when interpreting the findings. Although risk-of-bias and critical appraisal assessments were conducted, this scoping review was primarily intended to map the available literature. Therefore, the findings should be interpreted as a descriptive overview of the available evidence rather than as definitive evidence of intervention effectiveness. Moreover, all included studies were conducted in either China or the United States, which may limit the transferability of the findings to other healthcare systems and cultural contexts. Second, the literature search was limited to studies published within the past 5 years and was restricted to Chinese- and English-language databases, which may have led to the omission of relevant studies. Finally, this review included only RCTs and quasi-experimental studies; therefore, evidence from other study designs, including qualitative studies, may have been excluded. Future reviews could extend the search period, search additional databases and gray literature, and include other study designs to provide a more comprehensive understanding of discharge planning services for patients with diabetes.

## 5 Conclusion

In conclusion, this scoping review mapped the existing evidence on discharge planning services for patients with diabetes during the hospital-to-home transition. The included studies described discharge planning services as multicomponent interventions that may support continuity of care, commonly involving patient education, individualized guidance, follow-up support, medication management, lifestyle advice, and coordination among patients, caregivers, and healthcare providers. Although several common components were identified across the included studies, variations in delivery formats, outcome measures, follow-up periods, evaluation methods, and reporting quality limited the comparability, applicability, and interpretability of the available evidence. These findings provide a descriptive overview of existing approaches while highlighting areas where evidence remains limited or inconsistently reported. Taken together, they point toward the need for future research to establish clearly described, continuous, and adaptable discharge planning service models across different care contexts, with greater attention to intervention timing, duration, follow-up procedures, process evaluation, consistent outcome measures, stakeholder perspectives, and longer-term outcome assessment. Ultimately, these efforts may inform the development, implementation, and refinement of discharge planning services that are feasible, acceptable, and responsive to the diverse needs of individuals with diabetes across the hospital-to-home transition.

## Data Availability

The original contributions presented in the study are included in the article/Supplementary material, further inquiries can be directed to the corresponding authors.
